# Supplementary material for: High-rate intercity quantum key distribution with a semiconductor single-photon source
Source: Light Sci Appl. 2024 Jul 2;13:150. doi: 10.1038/s41377-024-01488-0 (PMC11219984; doi:10.1038/s41377-024-01488-0)
Supplement: Supplementary file 1 — Supplementary: High-rate intercity quantum key distribution with a semiconductor single-photon source [file 41377_2024_1488_MOESM1_ESM.pdf]

Supplementary: High-rate intercity quantum key distribution with a semiconductor single-photon source

Jingzhong Yang,[1] Zenghui Jiang,[1] Frederik Benthin,[1] Joscha Hanel,[1] Tom Fandrich,[1] Raphael Joos,[2] Stephanie Bauer,[2] Sascha Kolatschek,[2] Ali Hreibi,[3] Eddy Patrick Rugeramigabo,[1] Michael Jetter,[2] Simone Luca Portalupi,[2] Michael Zopf,[1] Peter Michler,[2] Stefan Kück,[3] and Fei Ding[1,4,*]

[1]*Institut für Festkörperphysik, Leibniz Universität Hannover, Appelstraße 2, 30167 Hannover, Germany*

[2]*Institut für Halbleiteroptik und Funktionelle Grenzflächen, Center for Integrated Quantum Science and Technology (IQST) and SCoPE, University of Stuttgart, Allmandring 3, 70569 Stuttgart, Germany*

[3]*Physikalisch-Technische Bundesanstalt, Bundesallee 100, 38116 Braunschweig, Germany*

[4]*Laboratorium für Nano- und Quantenengineering, Leibniz Universität Hannover, Schneiderberg 39, 30167 Hannover, Germany*

* fei.ding@fkp.uni-hannover.de

I. OVERVIEW OF THE EXPERIMENTAL SETUPS

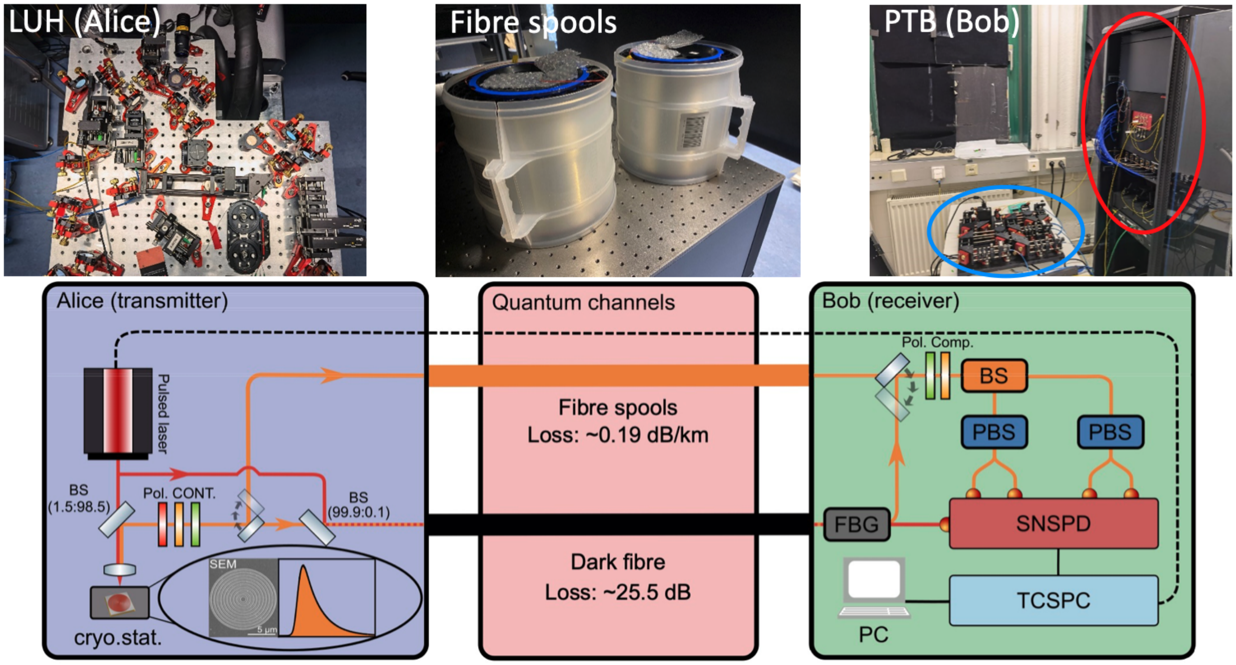

Fig. S1. Photographs (first row) and a schematic (second row) of our QKD system with a semiconductor quantum dot (QD) comprising the transmitter, fibre spools, receiver and super-conducting nanowire detectors (SNSPDs). The transmitter assembly (left) is located in the LUH laboratory and is positioned on the Attodry 1100 cryostat to facilitate QD excitation, polarisation state encoding and fibre coupling of single photon signals to either fibre spools or deployed fibres. A sequence of fibre spools (middle) is used to investigate the secret key rate (SKR) and quantum bit error ratio (QBER) dependent on transmission loss. In the QKD field experiment, the receiver module marked with a blue circle is located in the laboratory of PTB (right) in close proximity to the SNSPD from the company Single Quantum.

A. Transmitter efficiency

To calibrate the collection efficiency of our transmitter in Fig. S2 for the single-photons emitted by the quantum dot (QD), we transmit a power-stable laser of the same wavelength as the QD through the setup. We measure the laser powers using a powermeter (controller: Thorlabs PM100D, photodiode: S132C), after every optical element in the collection path of the transmitter

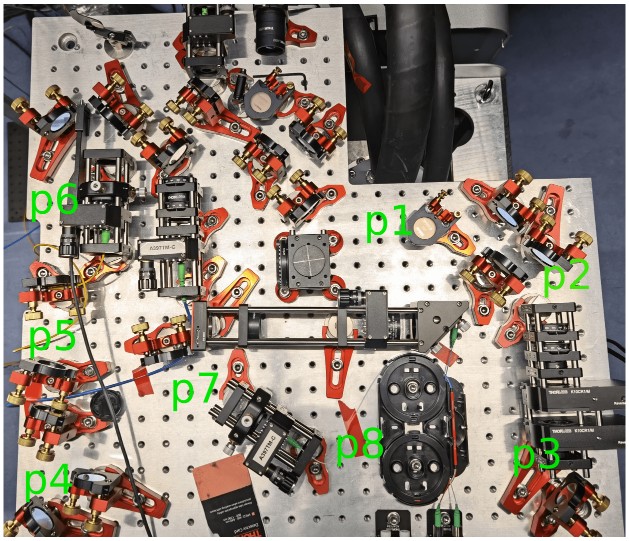

Fig. S2. Picture of the transmitter setup labelled with positions $p_1, p_2, ..., p_8$ for calibrating the efficiencies of optical components.

setup, as labelled from $p_1, p_2, ..., p_8$. In order to gain a more precise value, we collected data at each position for three minutes and calculated the average power.

B. Receiver efficiency

To estimate the overall transmission efficiency η of the receiver, we compare the power at the beginning of the optical setup with the sum of the power of all four possible optical paths through the setup. As a result, we obtain the calculated overall transmission efficiency,

$$\eta = \left(\sum_{i=1}^{4} P_{\text{out},i} \right) / P_{\text{in}} \tag{1}$$

Here, $P_{\text{out},i}$ denotes the power measured at the end of one of the four output ports indexed by i, through the single-mode fibre. Similarly, P_{in} represents the initial power measured at the beginning of the optical path in the receiver placed directly after the fibre which is connected to the laser. To make this measurement, a continuous wave diode laser (Thorlabs SFL1550P in a Thorlabs CLD1015 controller) is connected to a fibre-based 50:50 non-polarising beam splitter (BS). An output port of the BS connects to the photodiode of a polarimeter (Thorlabs PAX1000IR2) to measure the power before it reaches the receiver. The remaining port is connected through a single-mode fibre to the receiver. Another powermeter is used for calibrating the power measured by the photodiode of the polarimeter.

In each of the four optical paths of the receiver, the light passes through a polarising BS. In order to measure the free-space efficiency and fibre coupling efficiencies of the receiver, the input polarisation needs to be tuned. The linearly polarised light from the laser was set by use of the quarter waveplate (QWP) and half waveplate (HWP) at the beginning of the receiver, to match the polarisation of the nominally horizontal polarisation port. Thereafter, the power values in each of the four optical paths were recorded one after the other. Each time the power was measured for three minutes. The interval between data points was 0.1 s. The total efficiency was calculated using equation 1.

II. OPTICAL FIBRE CALIBRATION

A. Attenuation

1. Fibre spools

To quantify the average loss of the fibre spools (Corning SMF-28 Ultra, fixed length of 40.6 km for each) used in the laboratory, we calibrate the transmission loss as function of fibre length. As shown in Fig. **S3**, the telecom C-band CW laser (Thorlabs SFL1550P) is sent through a 50:50 fibre-based BS with one output port connected with a powermeter. The laser coming from the other output port is measured by a second powermeter after a sequence of fibre spools.

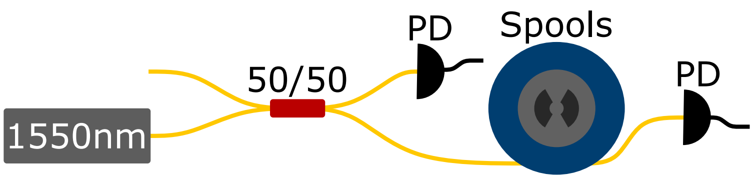

Fig. S3. Sketch of the experimental setup for measuring the average fibre spool losses. PD=Photodiode (both Thorlabs 132C). Measurement is implemented by reading the powers from two PDs concurrently.

The transmission losses through different number of fibre spools are measured and illustrated in Fig. **S4**. The average loss L_d is then extracted to be 0.1956 dB km^{-1} from the linear fitting. The existence of L_0 is attributed to losses at the connection between the fibre-based BS and the first fibre spool. The fitted value L_d is slightly higher than the value provided by the spool manufacturer (0.18 dB km), which is attributed to further losses at the connection interfaces between spools.

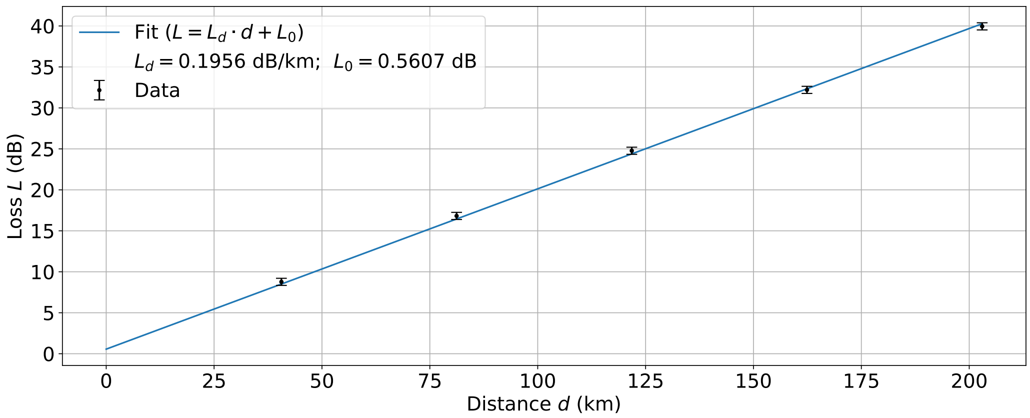

Fig. S4. Transmission loss as a function of the fibre spool length.

2. *Deployed fibre*

Similar to the average loss calibration setup for fibre spools, we measure the attenuation of the deployed fibre by substituting it for the fibre spools shown in Fig. S3. The power was measured for three minutes with a time interval of five seconds between data points. The attenuation in decibel is calculated as

$$\alpha = 10 \cdot \log_{10}\left(\frac{P_i}{P_f}\right) \tag{2}$$

where P_i and P_f are the initial and final power, that is before and after the deployed fibre, respectively. P_i and P_f are calculated as the mean of their respective measured data and their type A uncertainties are calculated as the experimental standard deviation of the mean. The uncertainty of the powermeters stated by the manufacturer was used as the type B uncertainty. The splitting ratio of the BS has been characterised and the deviation from a perfectly balanced BS is taken into account when calculating the mean power before the fibre. The resulting value of the attenuation is $(25.49 \pm 0.22)\,\mathrm{dB}$ by taking into account of the combined type A and B uncertainties [1, Chapter 5.1].

3. *Stability test*

To calibrate the attenuation stability of the deployed fibre that is essential for evaluating the stability of secret key transmission, we performed loss measurements using the same polarisation-

and power-stable CW laser and polarimeter as before. For each of the six polarisations $|A\rangle$, $|D\rangle$, $|H\rangle$, $|V\rangle$, $|R\rangle$ and $|L\rangle$, we measured the loss L through the fibre for three hours, and then examined the fluctuations of that loss about the respective average value L_{avg}. The results are shown in Fig. S5.

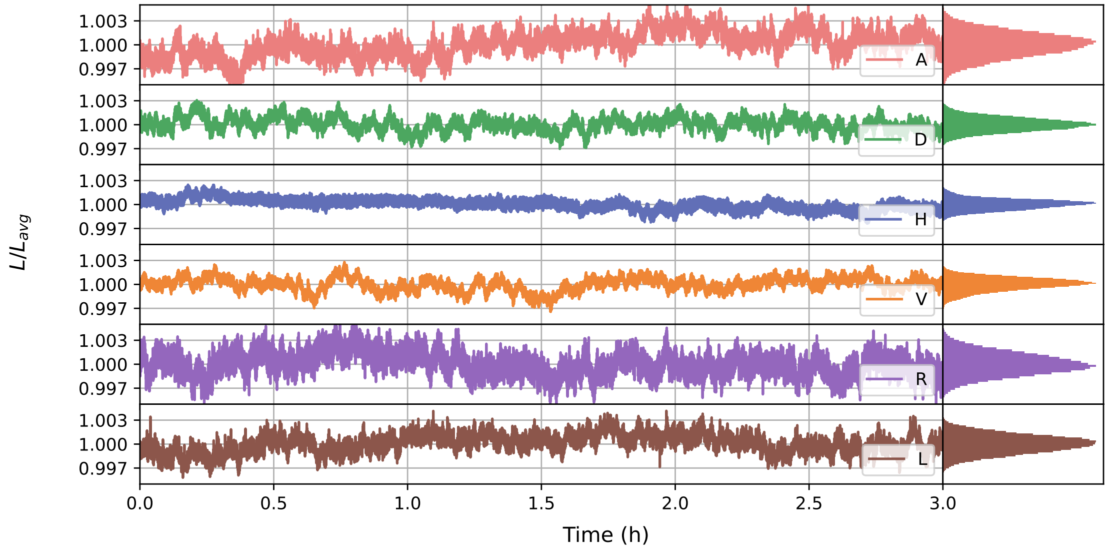

Fig. S5. Power stability over time for different polarisations. The polarisations referred to are those measured by Bob at the start of each measurement. For each measurement, the power variations remain on the sub-percent scale.

B. Polarisation Stabilisation

Parameter	Value
$\Delta\vartheta$	7°
QBER measurement time	1 s
Time between QBER checks	10 s
Maximum number of iterations	20
T_{QBER}	$0.1-2.0\%$

Tab. S1. Parameters used for the polarisation compensation algorithm.

As the deployed fibre used in the experiment runs through over $\sim 80\,\text{km}$, it is constantly subjected to varying levels and directions of stress due to changes in temperature, vibrations, or soil movements. To compensate for fluctuations in the polarisation due the fibre birefringence, an automatic feedback control loop was implemented. The QBER computed from counts on the SNSPD was used as the error signal, which was minimised whenever it crossed a given threshold, using the motorised polarisation controller (a pair of HWP and a QWP for our case). The compensation algorithm was applied to one basis at a time, and attempted to minimise the QBER for that basis. To determine the ideal angle by which to rotate the waveplates, the algorithm proposed in [2] was applied to both waveplates in an alternating fashion: the first and second derivatives of the QBER q with respect to the waveplate position ϑ are approximated as

$$\begin{aligned} \frac{\partial q}{\partial \vartheta} &\approx \frac{q(\vartheta+\Delta\vartheta)-q(\vartheta-\Delta\vartheta)}{2\Delta\vartheta}, \\ \frac{\partial^2 q}{\partial \vartheta^2} &\approx \frac{q(\vartheta+\Delta\vartheta)-2q(\vartheta)+q(\vartheta-\Delta\vartheta)}{(\Delta\vartheta)^2}, \end{aligned} \tag{3}$$

which requires three QBER measurements in total. The rotation to be applied to the waveplate is then

$$\sigma = \pm \frac{\partial q}{\partial \vartheta} \Big/ \frac{\partial^2 q}{\partial \vartheta^2}, \tag{4}$$

where the sign of σ is the opposite of that of the second derivative. Rotations were limited to $|\sigma| \leq 30^\circ$ to avoid overshooting.

For each QBER measurement, the counts C in the respective channels were accumulated over $1\,\text{s}$. Pre-measured detector dark counts were subtracted, and detection efficiencies were taken into account. The QBER was then calculated as

$$q = \frac{C_V}{C_V + C_H}, \tag{5}$$

here for the example of H-polarised input light. The above steps were looped until q fell below the given threshold, T_{QBER}. During data acquisition, it was checked regularly if a threshold T_{Check} threshold had been surpassed, and the compensation was initiated accordingly. We set $T_{\text{Check}} = T_{\text{QBER}} + 0.1\%$, to prevent small short-term or statistical fluctuations from triggering another compensation immediately after the previous one.

The compensation algorithm was executed on average about 10 (24) times per hour [3]. Compensations took from 30 seconds to, in rare cases, about 5 minutes. In total, 23% (39%) of the

measurement time was spent compensating polarisation fluctuations. We believe that this fraction can be reduced by at least one order of magnitude by optimizing algorithmic parameters and exchanging the slow rotation stages for a faster, more precise piezo-electronic fibre polarisation controller.

During some long-term measurements, it was found that the compensation algorithm occasionally struggled to go below the given QBER threshold, even if it had done so earlier during the same measurement. This is believed to be due to imprecise movement of the cage rotators and/or varying levels of unwanted daylight at the location of the detection setup. Therefore, an automatic ideal threshold search was implemented to determine the lowest possible threshold which was reliably achievable. This search was started each time the compensation algorithm failed to achieve the current threshold after a fixed number of iterations.

For this measurement, a fixed-polarisation signal was launched into the fibre by Alice, and the emerging polarisation was measured by Bob over an extended period of time (ca. three hours in this case). The signal consisted of a continuous wave laser at 1549 nm (Thorlabs SFL1550P in a Thorlabs CLD1015 controller), and the measurement was conducted using a polarimeter (Thorlabs PAX1000IR2). The laser emission had been previously checked to be highly stable in terms of power and polarisation over durations much longer than the measurement duration.

Figure S6 shows the result of this reference measurement, compared to a raw QBER measurement recorded during one of our data acquisition runs. It is evident that the polarisation compensation is working as intended, as the QBER is kept constantly low throughout the entire duration of the respective measurement. In the reference measurement without the compensation, the polarisation error E_{pol} continuously increases, which would lead to an analogous increase in the QBER. E_{pol} is computed from the projection of the Stokes vector at a time t, $\vec{S}(t)$, onto the Stokes vector at time $t = 0$:

$$E_{\mathrm{pol}}(t) = 1 - (\vec{S}(t) \cdot \vec{S}(0))^2 \tag{6}$$

III. CALIBRATION OF SNSPD

A. Channel efficiencies

To measure the efficiencies of the SNSPD channels, the photon flux of a weak quasi-monochromatic laser (Thorlabs SFL1550P in a Thorlabs CLD1015 controller) is calculated after measuring the

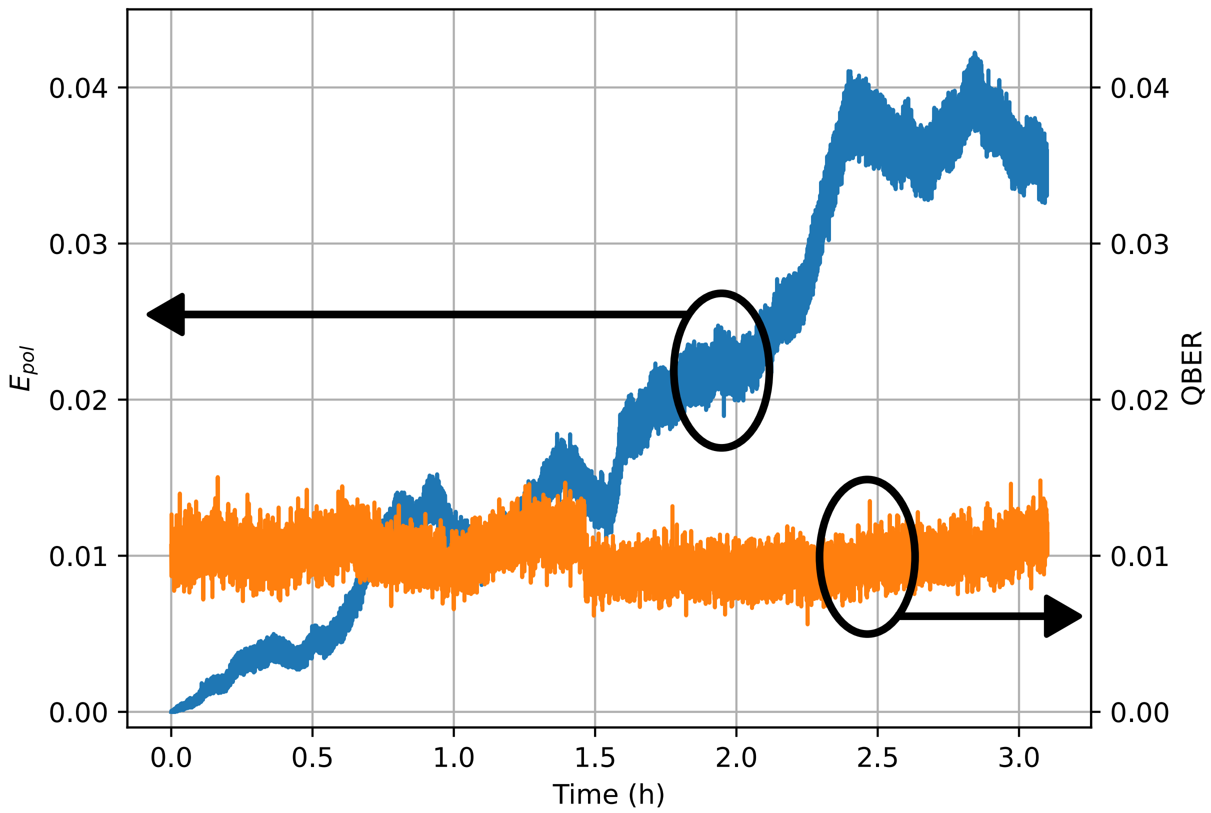

Fig. S6. Comparison of polarisation stability without (blue, left y-axis) and with (orange, right y-axis) automatic polarisation compensation enabled. In both cases, the initial polarisation was set such that Bob received H-polarised light at time $t = 0$.

beam's intensity using a powermeter. Using a known attenuation this laser beam has then been sufficiently attenuated in order to safely measure the photon flux using the single-photon detector. The system detection efficiency stated in the main text is the ratio of the measured and calculated photon flux.

In order to measure the intensity of the continuous wave laser the following setup is used: First the output beam is passed through neutral density filters and projected onto a linear polarisation using a polariser. This is used to adjust the intensity. Afterwards the beam traverses one pair of QWP and HWP before being coupled in to a single-mode fibre to a photodiode. These wave-plates are not yet rotated, but are already included for easier comparison. The light is then coupled into a single-mode fibre which connects to a powermeter. The power was recorded for three minutes with

a measurement interval of about 0.1 seconds, while the laser was turned on and off, respectively. These values P_{on} and P_{off} are used to calculate the estimated photon flux Φ via

$$\Phi = \frac{P_{\mathrm{on}} - P_{\mathrm{off}}}{h \cdot f} \tag{7}$$

where h is the Planck constant and f the light frequency. P_{on} and P_{off} are estimated as the mean value of the corresponding data. The uncertainties have been estimated as stated in chapter IV. OPTICAL FIBRE CALIBRATION using both type A and B measurement uncertainty evaluations. The uncertainty of the photon flux and in the following also the attenuation and the efficiency are calculated using Gaussian forward uncertainty propagation, that is by using a first-order Taylor series approximation [1, Chapter 5.1].

A fibre-based variable optical attenuator was then used to connect the single-mode fibre after the waveplates to the SNSPD to be characterised. The countrate averaged over one second was recorded for five minutes. The mean value is interpreted as the measured photon flux and the empirical standard deviation of the arithmetic mean as the measurement uncertainty.

The used attenuation of the fibre-based optical attenuator was measured separately using the continuous wave laser and a powermeter and calculated as $\alpha = 10 \cdot \log_{10}(P_{\mathrm{without}}/P_{\mathrm{with}})$, where P_{without} and P_{with} are the mean power values without and with the variable optical attenuator respectively. The uncertainties of P_{without} and P_{with} have been estimated as stated above using both type A and B measurement uncertainty evaluations. The value of the attenuation is $(56.13 \pm 0.31)\,\mathrm{dB}$. Using this value the detector efficiency is then calculated as

$$\eta = \frac{\Phi_m}{\Phi_c \cdot 10^{-\alpha/10}} \tag{8}$$

where Φ_c is the photon flux calculated using equation 7, Φ_m is the mean countrate measured using the single-photon detector and $\Phi_c \cdot 10^{-\alpha/10}$ is the estimated photon flux the detector should measure with perfect efficiency.

In the Physikalisch-Technische Bundesanstalt in Braunschweig four SNSPDs have been used together with the receiver for the field-based measurements. For the analysis of these measurements the mean value of the individual detector efficiencies for a wavelength of 1550 nm has been used. The individual values as given by the manufacturer are $(82 \pm 3)\,\%$, $(60 \pm 3)\,\%$, $(73 \pm 3)\,\%$, $(66 \pm 3)\,\%$. The mean value is $(70.3 \pm 1.5)\,\%$. With the uncertainty calculated using Gaussian forward uncertainty propagation. For the analysis the non-rounded value of 70.25 % has been used.

B. Dark count rate

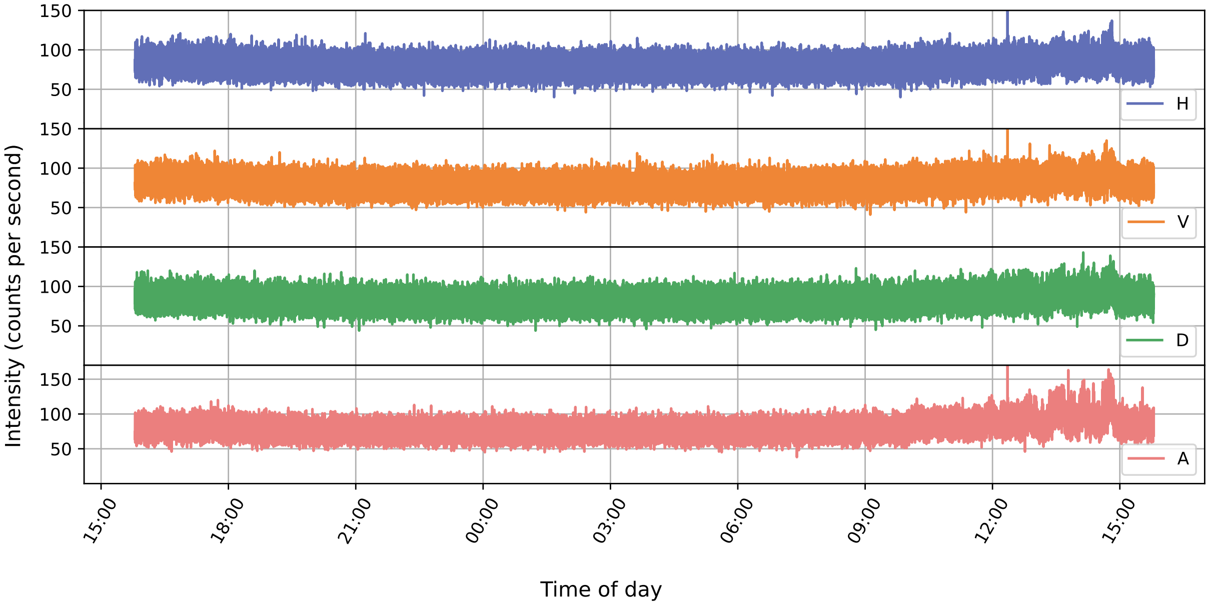

Fig. S7. Graph of the dark countrate over time for the 24 hour measurement that is used to calculate the dark countrate value used for the analysis of the field-based measurements. The labels $|H\rangle, |V\rangle, |D\rangle, |A\rangle$ indicate the polarisation that is associated with the optical path connected to the corresponding SNSPD. Near the end of the measurement there is a short peak of about 400 counts per second in the dark countrate that we associate with a brief change in the environment. The corresponding data points are outside the range of the ordinate of the graph and are not shown therein.

Only a single superconducting nanowire single-photon detector in the Leibniz University of Hannover was used for all the measurements, the dark countrate was measured for one channel by recording the time tags while the detector was connected to the setup without any laser turned on. The time tags were then analysed in order to calculate the countrate with a bin width of one second. The value of the dark countrate was calculated as the mean of these countrates averaged over one second and the uncertainty was estimated as the experimental standard deviation of the mean. The resulting value of the dark countrate is (49.80 ± 0.21) Hz.

In the Physikalisch-Technische Bundesanstalt in Braunschweig, the time tags of a 24-hour measurement were collected to calculate the dark countrates. During this measurement the four detectors were connected to the four optical paths of the receiver which in turn was connected to the deployed fibre. The other end of the deployed fibre in the Leibniz University of Hannover

was disconnected. The recorded time tags were then binned with a bin width of one second and the individual countrates were calculated as the mean with the experimental standard deviation of the mean as the associated uncertainty value. The four dark countrate values are: (80.420 ± 0.033) Hz, (80.007 ± 0.034) Hz, (82.591 ± 0.034) Hz, (79.64 ± 0.04) Hz. Because these values are very similar and out of convenience the mean of these four values (80.666 ± 0.018) Hz was used for the analysis of the field-based measurements. The associated uncertainty was calculated using Gaussian forward uncertainty propagation. The graph of the 24 h measurement of the dark countrate over the fibre is shown in Fig. S7.

IV. SOURCE CHARACTERISATION

A. Sample brightness

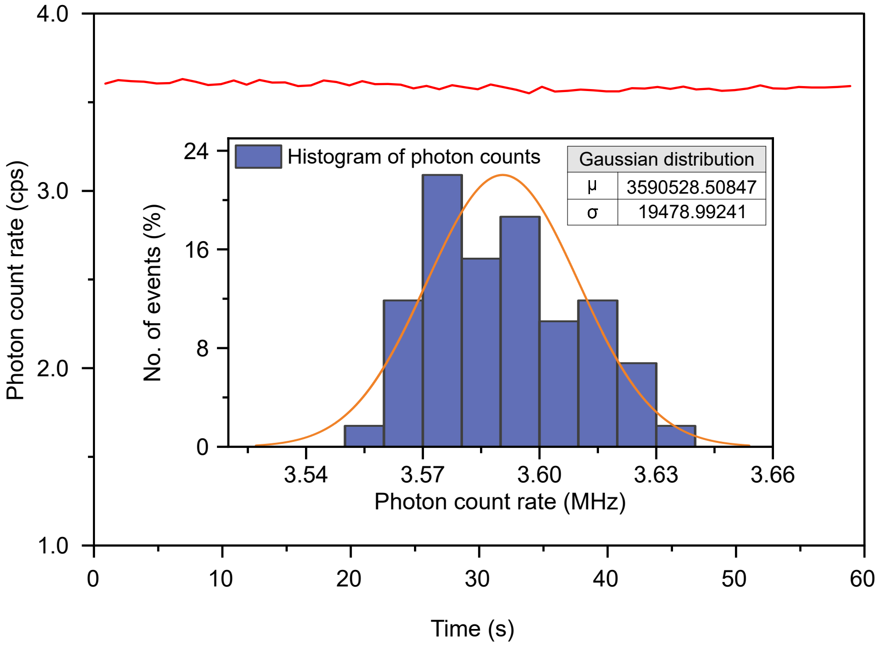

Fig. S8. Measurement of single-photon count rate from the transmitter as a function of time. The inset shows the statistical histogram (blue) of the photon count distribution that is fitted by the Gaussian distribution curve (orange curve)

B. Lifetime measurement

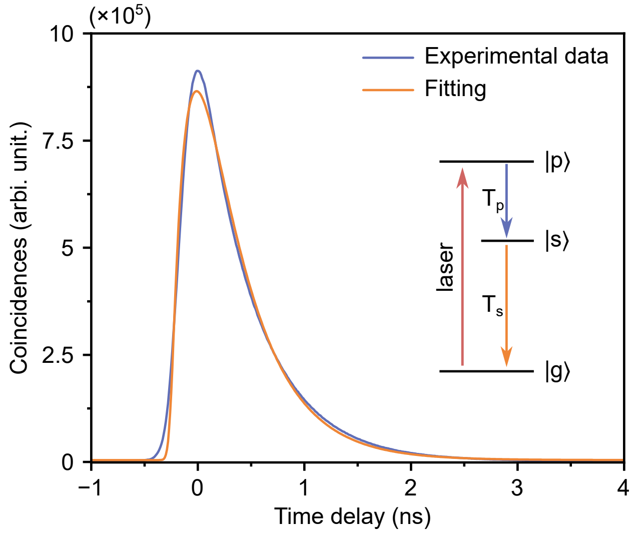

Fig. S9. Lifetime fitting. The blue curve represents the experimentally measured decay process, the orange curve represents the fitting results. Inset: The 3-level cascade system.

C. Blinking-corrected second-order auto-correlation

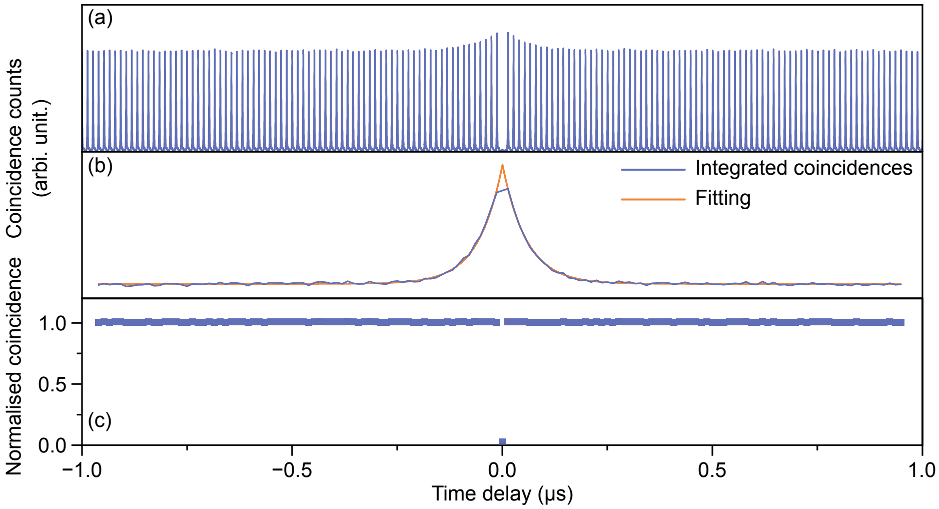

Fig. S10. Calculation of the blinking-corrected $g^{(2)}(0)$. (a) Exemplary second-order auto-correlation histogram. (b) Integrated coincidence counts (blue line) with respect to the peaks in (a) and no temporal filter applied. The orange line shows the blinking fitting function. (c) Normalised and blinking-corrected coincidence distribution as function of the time delay.

V. TRUTH TABLE MEASUREMENTS WITH THE DEPLOYED FIBRE

The histograms in the truth tables [Fig. 3(c) in the main text] present the lifetime measurements of our photon source for different input and output polarisations. Each histogram was computed by correlating the measured single-photon signal in the corresponding output channel with a reference signal.

The deployed fibre lifetime measurements for all four output ports could be executed concurrently because the SNSPD stationed at PTB has four channels with similar performance. Each row of the truth table in Fig. 3(c) of the main text represents a measurement where time tags for all four channels were recorded. The reference signal was composed of photons from a second output port of the excitation pulsed laser operating at approximately1530 nm. They were transmitted via the deployed fibre to a fifth channel on the same SNSPD. To decrease the laser power present in the fibre and avoid scattering-induced noise, a variable fibre-optical attenuator (FS VOA-B-FS) on Alice's side attenuated the laser below the saturation limit of the SNSPD.

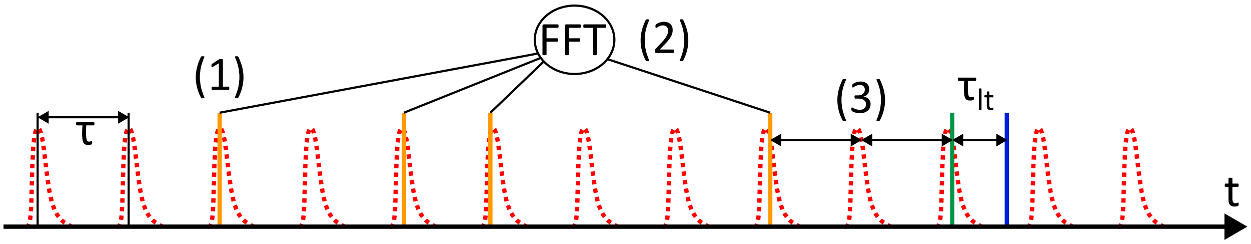

Fig. S11. Scheme of the correlation procedure executed in postprocessing. (1) A fraction of the laser pulses emitted by Alice (dashed red lines) is detected at certain timestamps by Bob (orange lines). (2) From these detection timestamps, Bob computes the time between two emitted pulses τ. (3) If Bob detects a signal photon (blue line), he can now infer the time at which he should have detected the most recent laser pulse (green line). For this, the most recently detected laser pulse is used as a reference point. Now, Bob can generate an according entry at τ_{lt} in his lifetime histogram.

During the experiment, the SNSPD reached a saturation point of around ~ 3 MHz and a reference signal was only measured for a portion of the excitation pulses, which had a CR of roughly 228 MHz. To still use all the measured counts for lifetime histograms, despite the imperfect reference signal, the following post-processing method was used: time tags were stored for all five channels, the laser CR was obtained using an FFT, and the missing laser time tags were artificially calculated and included with sub-picosecond precision. Figure S11 illustrates a sketch of

this process. Compared to standard synchronisation schemes that use more powerful laser pulses and fast photodiodes, this approach has the advantage of only needing a very weak laser signal in the transmission fibre. This significantly reduces any scattering effects that could reduce the final secure key rate. We observed no changes in the count rates of the four SNSPD signal channels when switching this reference signal on or off.

VI. ESTIMATION OF SECURE KEY RATE PARAMETERS

A. Click and error probability

Multi-photon emission is a threat for the security of QKD taking into account photon number splitting attacks. Here we evaluate the upper bound of multi-photon emission probability from our source as [4],

$$p_m \leq \frac{1 - ng^{(2)}(0) - \sqrt{1 - 2ng^{(2)}(0)}}{g^{(2)}(0)} \tag{9}$$

Considering the probability of n photon emissions $\{p_n\}$ from the QD photon source, in our experiment we assume the multiphoton contribution up to the 2-photon component to be negligible, i.e. $p_n = 0$ for $n \geq 3$. Therefore, the n-photon emission probability $\{p_n\}$ from the source can be simplified and calculated with the measured average photon number per pulse $\langle n \rangle$ and $g^{(2)}(0)$ [5, 6],

$$\begin{aligned} p_2 &= \frac{\langle n \rangle^2 \cdot g^{(2)}(0)}{2} \\ p_1 &= \langle n \rangle - p_2 \\ p_0 &= 1 - p_1 - p_2 \end{aligned} \tag{10}$$

The click probability of the n-photon number states on the receiver detectors are then given by [4, 5],

$$p_c^{(n)} = c_{dt} p_n \left[1 - (1 - p_{dc})(1 - \eta_T \eta_C \eta_R \eta_D)^n\right] \tag{11}$$

where η_T, η_C, η_R, η_D are the transmission efficiency of the transmitter, quantum channel, receiver and detector, respectively. p_{dc} is the dark count probability of the system. Assuming that the average dark count rate is d for each channel of the detector, we estimate $p_{dc} = 4 \cdot d \cdot \tau$ with 4 being the number of receiver ports and τ the temporal window size. c_{dt} is the correction factor taking into account the dead time of the detectors τ_{dt}, which can be modelled [5],

$$c_{dt} = \frac{1}{1 + R\tau_{dt} p_c^{X,Z}} \tag{12}$$

In the QKD scenario, the total click and error probability on the $\{X,Z\}$ bases are,

$$
\begin{aligned}
p_c^{X,Z} &= c_{dt}\sum_{n=0}^{2} p_n\left[1-\left(1-p_{dc}^{X,Z}\right)(1-\eta_T\eta_C\eta_R\eta_D)^n\right] \\
p_e^{X,Z} &= c_{dt}\left\{p_0 p_{dc}^{X,Z} + p_{mis}\cdot\sum_{n=1}^{2} p_n\left[1-\left(1-p_{dc}^{X,Z}\right)(1-\eta_T\eta_C\eta_R\eta_D)^n\right]\right\}
\end{aligned}
\tag{13}
$$

with parameter p_{mis} denoting the detection error probability caused by the optical misalignment of the whole system.

B. Estimation of bounds for QBER and detected single-photon fraction

For convenience, we assume the equal QBERs on $\{X,Z\}$ bases [4], which are defined for n-photon number states as,

$$e_n \equiv e_n^X \equiv e_n^Z = \frac{e_0 p_{dc} + p_{mis}\cdot[1-(1-\eta_T\eta_C\eta_R\eta_D)^n]}{1-(1-p_{dc})(1-\eta_T\eta_C\eta_R\eta_D)^n} \tag{14}$$

where $e_0 = 1/2$ indicates the error rate with the random background. The total QBER is given as,

$$e_{tot} = \frac{c_{dt}}{p_c}\sum_{n=0}^{2} e_n p_n\left[1-\left(1-p_{dc}^{X,Z}\right)(1-\eta_T\eta_C\eta_R\eta_D)^n\right] \tag{15}$$

In practice, the communication parties have to assume that all loss and errors come from the single states due to the untrusted channel parameters. It therefore gives out $e_{n\geq 2} = 0$ and the upper bound of QBER for a 1-photon state is [4],

$$\bar{e}_1 \leq \frac{e_{tot} p_c - e_0 p_0 p_{dc}}{1-(1-p_{dc})(1-\eta_T\eta_C\eta_R\eta_D)} \tag{16}$$

By taking the multi-photon emission probability into account, we can estimate the lower bound of detected 1-photon states [4],

$$\underline{p}_c^{(1)} \geq p_c - p_0 p_{dc} - p_m \tag{17}$$

C. Lower bound of sifted key block from non-multiphoton fraction

To determine the lower bound of the number of events contributed by non-multiphoton fraction, we first assume that all the expected number of multi-photon emissions from the single-photon source are detected by Bob ($n_{R,mp}^{X,Z*}$),

$$n_{R,mp}^{X,Z*} = n_{S,mp}^{X,Z} \approx n_S^{X,Z}\cdot p_m \tag{18}$$

where n_S is the finite block size of the keys sent by Alice, which is determined by the CR R and transmission time τ.

Following the analysis and notion in [7] for giving a more rigorous and optimal estimation on the deviation of statistical fluctuation, the upper Chernoff bound of the received multi-photon number on each basis is expressed as,

$$\overline{n}_{R,mp}^{X,Z} = (1+\delta^U)\cdot n_{R,mp}^{X,Z*} \tag{19}$$

where,

$$\delta^U = \frac{\beta + \sqrt{8\beta n_{R,mp}^{X,Z*} + \beta^2}}{2n_{R,mp}^{X,Z*}} \qquad \beta = \ln \varepsilon_{PE}^{-1} \tag{20}$$

Thus, the lower bound of the received keys from the non-multiphoton fraction on each basis is,

$$\underline{n}_{R,nmp}^{X,Z} = n_R^{X,Z} - \overline{n}_{R,mp}^{X,Z} \tag{21}$$

with $n_R^{X,Z} = {}^{n_{S,mp}^{X,Z}}/{}_{p_c^{X,Z}}$.

D. Upper bound of phase error rate

For practical QKD, Alice and Bob preferentially choose biased projection basis for a more efficient sifting of the keys. In our work, we follow the convention of defining the rectilinear polarisation as the Z basis, and diagonal polarisation as the X basis. The Z basis will be used to generate the keys of bits for information communication, while all the keys announced publicly on the X basis will be used to evaluate the information leakage to an eavesdropper from the Z basis. The phase error rate in the Z basis ϕ_z could be conservatively estimated based on the received non-multiphoton fraction in the X basis $\underline{n}_{R,nmp}^{X}$,

$$\phi^Z = \frac{m^X}{\underline{n}_{R,nmp}^{X}} \tag{22}$$

in which m_X is the number of errors on the X basis $m^X = N_S^X \cdot p_e^X$.

Equation 22 shows the estimate of the phase error rate in the Z basis ϕ^Z using the result from the X basis. The upper bound of the phase error rate in the X basis under the assumption that $0 < \phi^Z < \overline{\phi}^Z < 1$ can be calculated with random sampling without replacement [5, 7],

$$\overline{\phi}^Z = \phi^Z + \gamma^U(N_R^Z, N_R^X, \phi^Z, \varepsilon) \tag{23}$$

where,

$$\gamma^{U}(n,k,\lambda,\varepsilon)=\frac{\frac{(1-2\lambda)AG}{n+k}+\sqrt{\frac{A^2G^2}{(n+k)^2}+4\lambda(1-\lambda)G}}{2+2\frac{A^2G}{(n+k)^2}},$$
$$A=max\{n,k\},$$
$$G=\frac{n+k}{nk}\cdot\ln\frac{n+k}{2\pi nk\lambda(1-\lambda)\varepsilon^2}, \tag{24}$$

and ε is the common failure probability for ε_{sec}-security ($\varepsilon_{sec}=\varepsilon_{PA}+\varepsilon_{PE}+\varepsilon_{EC}$) [8], in which $\varepsilon_{PA}=\varepsilon$ and $\varepsilon_{PE}=2n_{PE}\varepsilon$. The number of quantified constraints in post-processing is $n_{PE}=2$.

E. Lower bound of error correction leakage in one-way protocols

In the finite key regime, we employ the improved approximation method on the information leakage during the one-way information reconciliation [9], expressed as,

$$\lambda_{ec}\geq n_R^Z\cdot h(e_{tot})+\left[n_R^Z(1-e_{tot})-F^{-1}\left(\varepsilon_{cor}\cdot\left(1+\frac{1}{\sqrt{n_R^Z}}\right);n_R^Z,1-e_{tot}\right)-1\right]\cdot\log_2\frac{1-e_{tot}}{e_{tot}}$$
$$-\frac{1}{2}\log_2 n_R^Z-\log_2\frac{1}{\varepsilon_{cor}} \tag{25}$$

where $F^{-1}\left(\varepsilon_{cor}\left(1+1/\sqrt{n_R^Z}\right);n_R^Z,1-e_{tot}\right)$ is the inverse of the cumulative distribution function of the binomial distribution.

F. Emulation of QKD performance with further optimisation

In Fig. 4 of the main text, we estimate the figure of merit of the QKD performance using further optimised single-photon source and detection systems. The emulation of finite SKBs per pulse is implemented by using the experimentally achievable source quality of optimised emitters at shorter wavelengths (brightness, $g^2(0)$), detector system and the asymmetric BB84 protocol. The detailed parameters are listed in the following Tab.S2,

The telecom C-band SPS is assumed to be measured in a Fabry-Perot microcavity system with a concave top mirror structure coupling the photons into the near-field fibre [10]. The use of the gate bias applied to the QD enables the high excitation efficiency of up to 0.96 (average number of photons per pulse). The reported efficiency of 0.86 for specific polarisation generation can be further improved to near unity by a stimulation scheme in a quantum ladder system [13, 14]. The

Tab. S2. Experimentally achievable parameters of the QKD system with SPS based on QDs

Description	Parameter	Value
Purcell factor	F_p	12 [10]
Average photon number per pulse	$\langle n \rangle$	0.96 [10]
Probability of emission into H-polarised mode	η_H	0.86 [10]
Clock rate	R	1 GHz [11]
Second-order correlation	$g^{(2)}(0)$	0.0075 % [12]
Transmitter efficiency	η_T	0.69 [10]
EOM efficiency	η_{eom}	0.643 [a]
EOM error ratio	e_{eom}	0.312 % [a]
Receiver efficiency	η_R	0.740 [b]
Detector efficiency	η_D	0.820 [10]
System misalignment probability	p_{mis}	6.302×10^{-3} [c]
Dark count per second	R_{dc}	0.03 [11]
Averaged fibre-spool loss	l	$0.1956\,\mathrm{dB\,km^{-1}}$ [b]
Finite-block size	n_R^Z	10^8 [b]
Parameter estimation failure probability	ε_{PE}	$2\times10^{-10}/3$ [b]
Error correction failure probability	ε_{EC}	$10^{-10}/6$ [b]
Privacy amplification failure probability	ε_{PA}	$10^{-10}/6$ [b]
Error verification failure probability	ε_{cor}	10^{-15} [b]
Error correction leakage	f_{EC}	1.16 [b]

[a] The efficiency and extinction ratio of the EOM have been characterised in VIII A and VIII B, respectively

[b] Default values used in this manuscript.

[c] Evaluated according to the average QBER that is measured under the active modulation with the EOM

Purcell factor of 12 ensures the excitation CR (CR) up to 1 GHz without CR saturation. Furthermore, the GaAs/AlGaAs QDs have achieved the record value of $g^{(2)}(0) = (7.5 \pm 1.6) \times 10^{-5}$ of second-order autocorrelation at zero delay. Apart from the source, the quality of the quantum channel and the detection system are the limiting factors for QKD performance. The dark count rates of 0.01 Hz and 0.02 Hz for the quantum channel and the superconducting nanowire detectors,

respectively, have been demonstrated in the field [11]. For the QKD with SPS, the influence of the multi-photon fraction $\sim \langle n \rangle^2 g^2(0)/2$ as a function of the average number of photons per pulse $\langle n \rangle$ is of more significance than the single-photon fraction $\langle n \rangle$. An optimal pre-attenuation for $\langle n \rangle$ is therefore useful to suppress the multi-photon fraction in order to obtain higher SKBs per pulse. In this experiment, we implemented the standard BB84 protocol with two balanced (X and Z) projection bases, that is $p_X = \frac{1}{2}$. It is known that using asymmetric bases choice can improve the secret key rate without compromising security [15]. One basis is used for key generation and the other for error estimation. For each loss shown in Fig. 4, we maximised the SKBs per pulse by dynamically optimising the best numerical solution for $0 < p_X < 1$ and L_{att}.

G. Temperature-dependent secret key rate per pulse

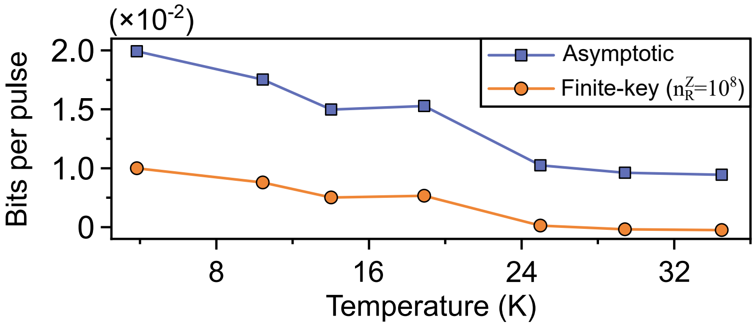

Fig. S12. Secret key per clock pulse as a function of temperature in the asymptotic and finite-key regime with block size of $n_R^Z = 10^8$.

For real-world quantum applications involving QDs, it is technically important to investigate their performance at high cryogenic temperatures (>4 K). This is because constructing a compact QD-based system is complex and the cryogenic temperatures typically require bulky compressors. In our experiment, we studied the temperature-dependent SKBs per pulse as shown in Fig. **S12**. The values degrade by approximately 50 % when the temperature is tuned from 4 K to 34 K. This is mainly due to reduced brightness and single photon purity [16]. Despite this, the overall performance remains well-preserved, making it feasible to employ compact cryogenic cooling systems for commercial plug-and-play QKD systems.

VII. MAXIMISATION OF SKR WITH 2D- TEMPORAL FILTER

The performance of the QKD can be enhanced utilising temporal filtering of the photon arrival time probability distribution [17]. Exemplary, the quantum bit error ratio (QBER) of the H-channel is calculated with $\mathrm{QBER_H}(\Delta t, t_c) = N_V(\Delta t, t_c)/(N_H(\Delta t, t_c) + N_V(\Delta t, t_c))$ where $N_H(N_V)$, Δt and t_c represent the number of coincidences with the H(V)-channel, the acceptance window width and the centre position of the window respectively. By reducing the temporal window Δt, a reduction of noise contributions from dark counts in the photon correlations can be observed resulting in a larger signal-to-noise ratio and lower QBER [17]. In consequence, a smaller acceptance window leads to fewer integrated coincidences in the detection channels impairing the number of received quantum bits. Thus an optimal window width can be determined with simultaneous low error ratios and a large number of bits ensuring an overall higher secret key rate. Due to the asymmetric arrival time distribution, the centre position of the acceptance window has to be refined to receive an optimal secure key rate S as a function of the two-dimensional parameter space $(\Delta t, t_c)$. The normalised secure key rate $S(\Delta t, t_c)$ is shown in the upper panel of Fig. **S13** as a heat map. The circle indicates the optimal window width and centre position at which the largest secure key rate is obtained. The lower panel of Fig. **S13** shows the photon arrival time distribution for different output polarisation states.

A key figure of merit for the security in QKD is the single-photon character of the emission. This property of the emission is manifested in the value of the second-order intensity autocorrelation at zero time delay $g^{(2)}(0)$. For the calculation of the asymptotic secure key rate, it is assumed that the blinking-corrected $g^{(2)}(0)$ value is constant over the acquisition time during the transmission. The blinking-corrected $g^{(2)}(0)$ value is derived from the accumulated coincidence counts over the whole acquisition time at the end of the measurement. The width of the time window for the integrated coincidence counts is set to 4400 ps which matches the magnitude of the inverse laser repetition rate.

The calculated blinking-corrected $g^{(2)}(0)$ values for the $|H\rangle$, $|V\rangle$, $|D\rangle$, $|A\rangle$ polarised input state are (0.057 ± 0.004), (0.0545 ± 0.0016), (0.0562 ± 0.0025), (0.082 ± 0.005), respectively. For the finite secure key rate per 30 min, the coincidence counts in the second-order correlation is accumulated likewise for 30 min. After every 30 minutes acquisition time, the blinking-corrected $g^{(2)}(0)$ value is calculated and utilised for the derivation of the finite secure key rate. The width of the time window for the integrated coincidence counts in the blinking-corrected $g^2(0)$ is set to

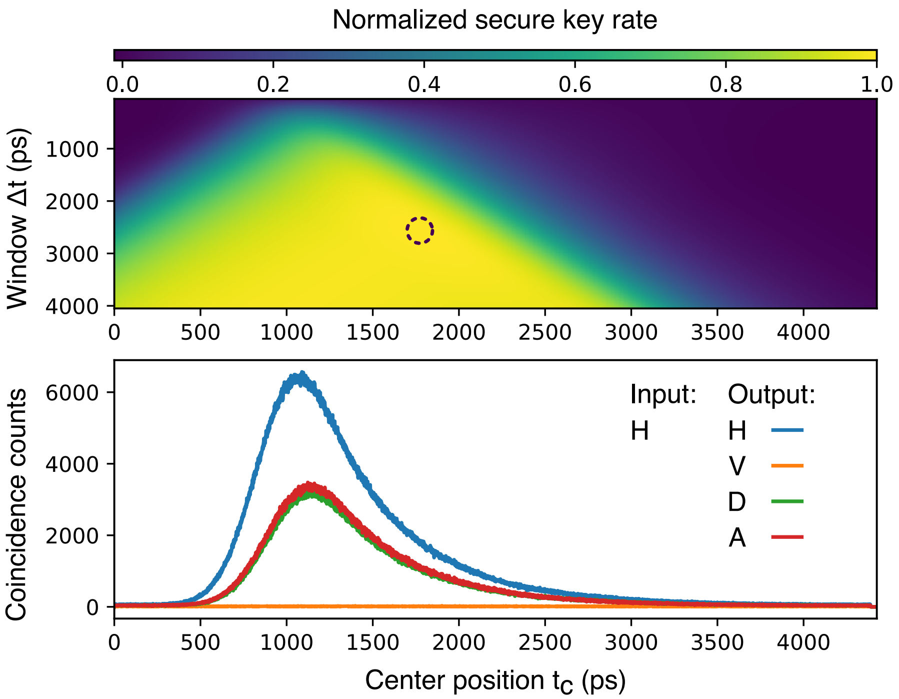

Fig. S13. Exemplary optimal secure key rate is obtained at a window size of $\Delta t = 2550\,\mathrm{ps}$ and center position $t_c = 1750\,\mathrm{ps}$. The circle (purple) marks these parameters in the heat map in the upper panel. The photon arrival time distributions in the lower panel were accumulated for 10 minutes with a binning size of 1 ps. The four different output polarisation states H (blue), V(orange), D (green), and A (red) are shown for the H-polarised input state.

be equal to the temporal window Δt of the 2D filtering. Thus the blinking-corrected second-order correlation at zero time delay and the normalised secure key rate can be written as $g^2(0,\Delta t)$ and $S(\Delta t, t_c, g^{(2)}(0,\Delta t))$, respectively.

VIII. TEST OF THE ACTIVE MODULATION ON POLARISATION

In the real-world QKD with BB84 protocol, the polarisation states are required to be sent randomly from the transmitter (Alice). The keys are then spontaneously shared between Alice and Bob by comparing the choice of measurement bases. A common approach for achieving the random encoding of each qubit is to employ the electron-optical modulator (EOM), that usually introduce additional optical loss. Additional errors with the polarisation are caused by the limited extinction ratio of the EOM. Besides, the necessity of high-speed switch of voltage applied on the EOM causes the distortion of voltage control, leading to the additional errors. To identify

these influence on the performance of our QKD, we implement a proof-of-the-concept experiment by involving the active modulation on the polarisation states. A high-speed polarisation switcher (PSW-LN-0.1-P-P-FA-FA from iXblue company) connected between the transmitter and receiver, is electronically controlled by the Field Programmable Gate Arrays (FPGAs) which is synchronised with the excitation laser source under a CR of 75.947 MHz.

A. Efficiency of the EOM

Similar as the setup for the fibre spools transmission characterisation (Fig. S3 in Sec. II A 1), here we calibrate the transmission of EOM by recording the powers from the one channel of BS and the other channel from the EOM over 8 h, simultaneously. According to Fig. S14, the average transmission efficiency of the EOM is approximately 0.643, equivalent to a loss of $\sim$1.938 dB.

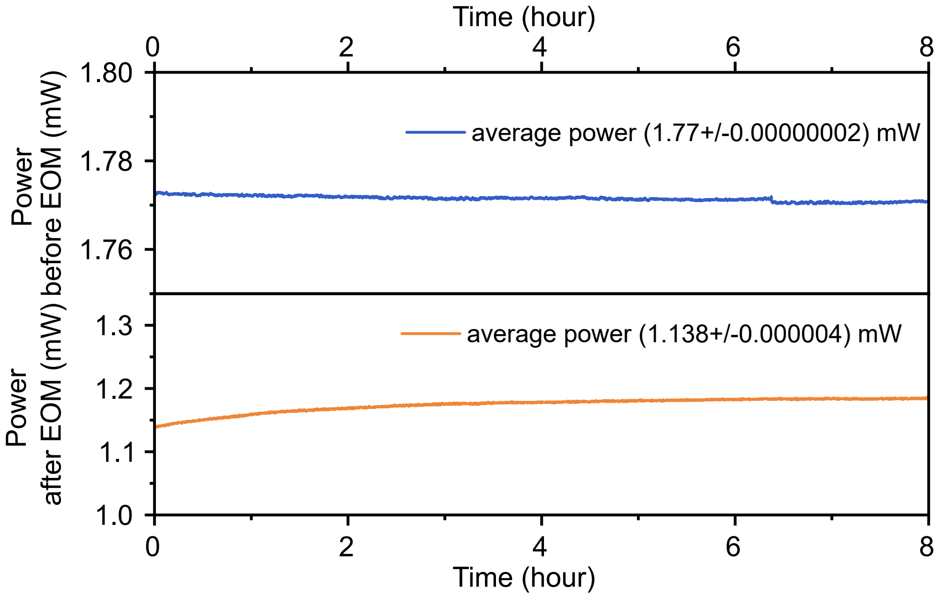

Fig. S14. Optical power before and after the EOM as a function of time.

B. Extinction ratio of the EOM

The extinction ratio of the EOM is calibrated by the fibre-based Extinction ratio meter (Thorlabs ERM200), by coupling the polarised laser after the Thorlabs film polariser (LPNIR050-MP2) to the EOM. A list of extinction ratio values measured every 30 s and shown in the following table, from which we calibrate the average extinction ratio of (25.055 ± 0.032) dB considering

the type-A uncertainty evaluation ($t_p = 1.06$). This corresponds to an additional error ratio of (0.312 ± 0.002) %.

Tab. S3. Extinction ratio of fibre-based EOM

No.	1	2	3	4	5	6	7	8	9	10
Extinction ratio (dB)	25.7	25.4	25.3	25.0	24.8	24.7	24.5	24.8	25.1	25.3

C. Impact of the active modulation

Apart from the loss and degradation of the extinction ratio caused by the EOM, the high-speed generation of quantum states from the EOM manipulated with random voltages poses a challenge to the accuracy of the electronic control. To systematically investigate the effect of the active modulation on our QKD system in terms of QBER, we calibrate the system by sending the CW laser through the transmitter, EOM, receiver to the detectors. A pre-defined 32-bit random sequence of bi-voltages from the FPGA is applied to the EOM with a CR of 75.947 MHz to randomly encode the polarisation states into $|V\rangle$ and $|A\rangle$. Normalised coincidence histograms between the clock and the four ports of the receiver are measured in the top two graph of Fig. S15. We extract the the QBERs from the X, Z bases as a function of time delay (Fig. S15). An averaged QBER of (0.63 ± 0.26) % is obtained while setting the temporal window of 4 ns. This QBER is eventually fed into the calculation of the misalignment probability p_{mis} of our QKD system.

IX. COMPARISON WITH OTHER QKD EXPERIMENTS

Figure S16 shows a comparison of the performance of our system with previous QKD experiments. It can be viewed as an extension of Fig. 4 in the main text. The lower figure shows the same data as the upper one, zoomed in on the range of $0 - 40$ dB. It is evident that in terms of the secret key rate per pulse, our system shows the best performance achieved with true single photons to date over a wide range, and is even competitive with state-of-the-art implementations using weak coherent pulses.

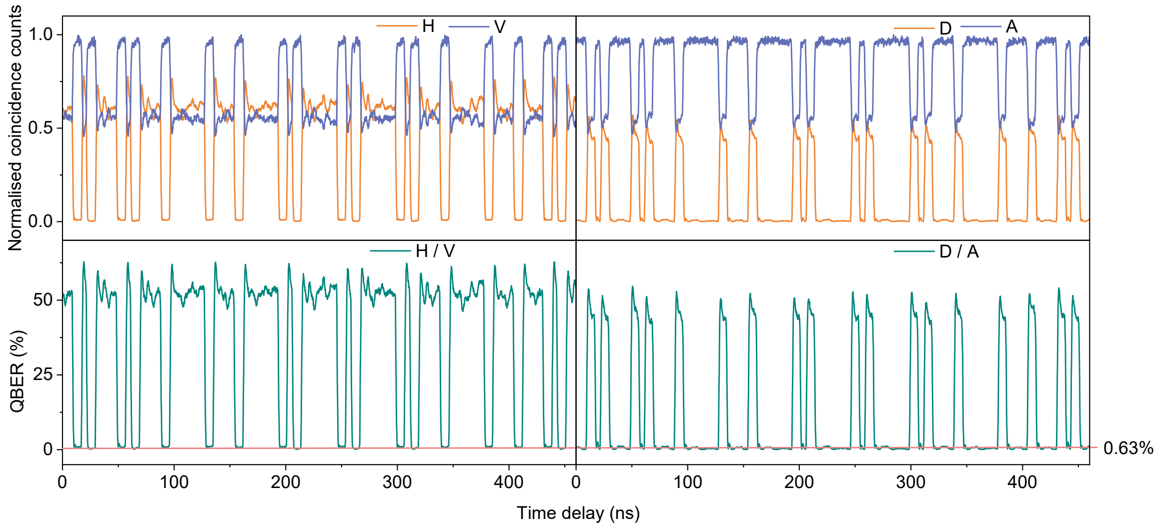

Fig. S15. Test the QBER of our QKD system using the active modulation. The polarisation of the CW laser passing through the whole system is repeatedly encoded between $|V\rangle$ and $|A\rangle$ with a 32-bit random binary sequence. The top two graphs shows the normalised histograms of the coincidences on X,Z bases. The extracted QBER from the bases as function of the encoding time are shown in the lower two figures.

[1] BIPM, IFCC., IFCC, ILAC., ISO, IUPAC. & IUPAP, OIML. Evaluation of measurement data—guide to the expression of uncertainty in measurement, JCGM 100: 2008 GUM 1995 with minor corrections. *Joint Committee for Guides in Metrology* **98** (2008).

[2] Mekhtiev, E. E., Gerasin, I. S., Rudavin, N. V., Duplinsky, A. V. & Kurochkin, Y. V. Polarization control algorithm for QKD systems. *Journal of Physics: Conference Series* **2086**, 012092 (2021).

[3] As the measurement for D-polarised input light performed exceptionally poorly in terms of polarisation stability, it is excluded in the average. Performance with this measurement included is specified separately in parentheses.

[4] Vyvlecka, M. *et al.* Robust excitation of c-band quantum dots for enhanced quantum communication (2023).

[5] Morrison, C. L. *et al.* Single-emitter quantum key distribution over 175 km of fibre with optimised finite key rates. *Nature Communications* **14**, 3573 (2023).

[6] Bozzio, M. *et al.* Enhancing quantum cryptography with quantum dot single-photon sources. *npj Quantum Information* **8**, 104 (2022).

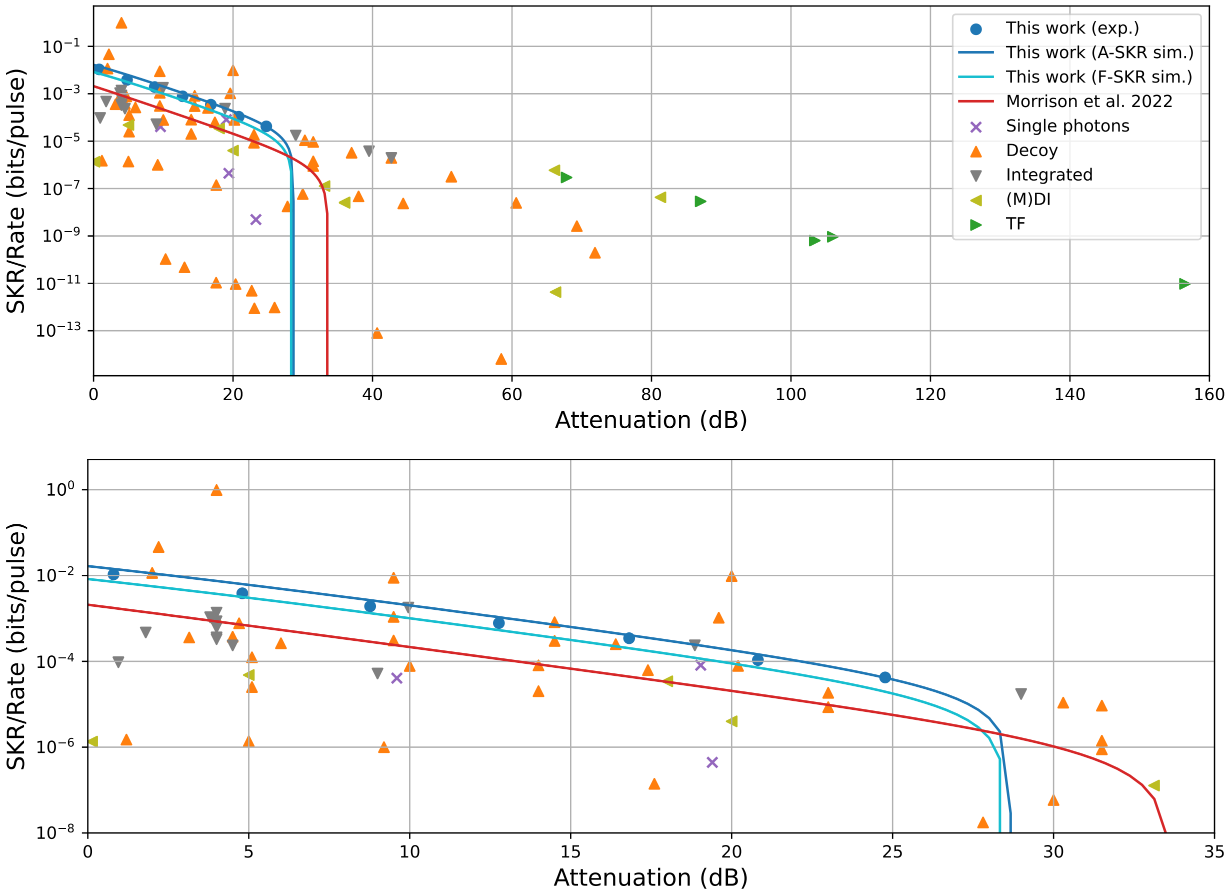

Fig. S16. Performances of our QKD system and previously reported QKD experiments. The performance of our system is measured by the finite SKR for a block size of 10^8; the other datapoints represent a mix of asymptotic SKRs and finite SKRS of different block sizes. SPS experiments: [5, 18–20], decoy state experiments: [21–45], integrated photonics experiments: [46–53], MDI/DIQKD experiments: [54–59], TFQKD experiments: [11, 60, 61].

[7] Yin, H.-L. *et al.* Tight security bounds for decoy-state quantum key distribution. *Scientific Reports* **10**, 14312 (2020).

[8] Bunandar, D., Govia, L. C. G., Krovi, H. & Englund, D. Numerical finite-key analysis of quantum key distribution. *npj Quantum Information* **6** (2020).

[9] Tomamichel, M., Martinez-Mateo, J., Pacher, C. & Elkouss, D. Fundamental finite key limits for one-way information reconciliation in quantum key distribution. *Quantum Information Processing* **16**, 280 (2017).

[10] Tomm, N. *et al.* A bright and fast source of coherent single photons. *Nature Nanotechnology* **16**, 399–403 (2021).

[11] Liu, Y. *et al.* Experimental twin-field quantum key distribution over 1000 km fiber distance. *Physical Review Letters* **130**, 210801 (2023).

[12] Schweickert, L. *et al.* On-demand generation of background-free single photons from a solid-state source. *Applied Physics Letters* **112** (2018).

[13] Wei, Y. *et al.* Tailoring solid-state single-photon sources with stimulated emissions. *Nature Nanotechnology* **17**, 470–476 (2022).

[14] Sbresny, F. *et al.* Stimulated generation of indistinguishable single photons from a quantum ladder system. *Physical Review Letters* **128**, 093603 (2022).

[15] Lo, H.-K., Chau, H. & Ardehali, M. Efficient quantum key distribution scheme and a proof of its unconditional security. *Journal of Cryptology* **18**, 133–165 (2004).

[16] Nawrath, C. *et al.* Bright source of purcell-enhanced, triggered, single photons in the telecom c-band. *Advanced Quantum Technologies* **6** (2023).

[17] Kupko, T. *et al.* Tools for the performance optimization of single-photon quantum key distribution. *npj Quantum Information* **6** (2020).

[18] Zahidy, M. *et al.* Quantum key distribution using deterministic single-photon sources over a field-installed fibre link. *npj Quantum Information* **10** (2023).

[19] Yuan, Z. L., Sharpe, A. W. & Shields, A. J. Unconditionally secure one-way quantum key distribution using decoy pulses. *Applied Physics Letters* **90** (2007).

[20] Takemoto, K. *et al.* Quantum key distribution over 120 km using ultrahigh purity single-photon source and superconducting single-photon detectors. *Scientific Reports* **5** (2015).

[21] Wang, S. *et al.* Practical gigahertz quantum key distribution robust against channel disturbance. *Optics Letters* **43**, 2030 (2018).

[22] Dixon, A. R. *et al.* High speed prototype quantum key distribution system and long term field trial. *Optics Express* **23**, 7583 (2015).

[23] Fröhlich, B. *et al.* Long-distance quantum key distribution secure against coherent attacks. *Optica* **4**, 163 (2017).

[24] Lucamarini, M. *et al.* Efficient decoy-state quantum key distribution with quantified security. *Optics Express* **21**, 24550 (2013).

[25] Sasaki, M. *et al.* Field test of quantum key distribution in the tokyo QKD network. *Optics Express* **19**, 10387 (2011).

[26] Liu, Y. *et al.* Decoy-state quantum key distribution with polarized photons over 200 km. *Optics Express* **18**, 8587 (2010).

[27] Dixon, A. R., Yuan, Z. L., Dynes, J. F., Sharpe, A. W. & Shields, A. J. Gigahertz decoy quantum key distribution with 1 mbit/s secure key rate. *Optics Express* **16**, 18790 (2008).

[28] Li, W. *et al.* High-rate quantum key distribution exceeding 110 mb s–1. *Nature Photonics* **17**, 416–421 (2023).

[29] Lu, F.-Y. *et al.* Experimental demonstration of fully passive quantum key distribution (2023). 2304.11655.

[30] Tang, B.-Y. *et al.* Free-running long-distance reference-frame-independent quantum key distribution. *npj Quantum Information* **8** (2022).

[31] Wang, K. *et al.* Round-robin differential phase-time-shifting protocol for quantum key distribution: Theory and experiment. *Physical Review Applied* **15**, 044017 (2021).

[32] Grünenfelder, F., Boaron, A., Rusca, D., Martin, A. & Zbinden, H. Performance and security of 5 GHz repetition rate polarization-based quantum key distribution. *Applied Physics Letters* **117**, 144003 (2020).

[33] Wang, S. *et al.* Beating the fundamental rate-distance limit in a proof-of-principle quantum key distribution system. *Physical Review X* **9**, 021046 (2019).

[34] Liu, H. *et al.* Experimental 4-intensity decoy-state quantum key distribution with asymmetric basis-detector efficiency. *Physical Review A* **100**, 042313 (2019).

[35] Bunandar, D. *et al.* Metropolitan quantum key distribution with silicon photonics. *Physical Review X* **8**, 021009 (2018).

[36] Boaron, A. *et al.* Secure quantum key distribution over 421 km of optical fiber. *Physical Review Letters* **121**, 190502 (2018).

[37] Yuan, Z. L., Dixon, A. R., Dynes, J. F., Sharpe, A. W. & Shields, A. J. Practical gigahertz quantum key distribution based on avalanche photodiodes. *New Journal of Physics* **11**, 045019 (2009).

[38] Rosenberg, D. *et al.* Practical long-distance quantum key distribution system using decoy levels. *New Journal of Physics* **11**, 045009 (2009).

[39] Dixon, A. R., Yuan, Z. L., Dynes, J. F., Sharpe, A. W. & Shields, A. J. Gigahertz decoy quantum key distribution with 1 mbit/s secure key rate. *Optics Express* **16**, 18790 (2008).

[40] Wang, Q. *et al.* Experimental decoy-state quantum key distribution with a sub-poissionian heralded single-photon source. *Physical Review Letters* **100**, 090501 (2008).

[41] Zhen-Qiang, Y. *et al.* Experimental decoy state quantum key distribution over 120 km fibre. *Chinese Physics Letters* **25**, 3547–3550 (2008).

[42] Rosenberg, D. *et al.* Long-distance decoy-state quantum key distribution in optical fiber. *Physical Review Letters* **98**, 010503 (2007).

[43] Peng, C.-Z. *et al.* Experimental long-distance decoy-state quantum key distribution based on polarization encoding. *Physical Review Letters* **98**, 010505 (2007).

[44] Zhao, Y., Qi, B., Ma, X., Lo, H.-K. & Qian, L. Experimental quantum key distribution with decoy states. *Physical Review Letters* **96**, 070502 (2006).

[45] Cao, Y. *et al.* Long-distance free-space measurement-device-independent quantum key distribution. *Physical Review Letters* **125**, 260503 (2020).

[46] Sibson, P. *et al.* Integrated silicon photonics for high-speed quantum key distribution. *Optica* **4**, 172 (2017).

[47] Sax, R. *et al.* High-speed integrated QKD system. *Photonics Research* **11**, 1007 (2023).

[48] Wei, K. *et al.* Resource-efficient quantum key distribution with integrated silicon photonics. *Photonics Research* **11**, 1364 (2023).

[49] Ma, C. *et al.* Silicon photonic transmitter for polarization-encoded quantum key distribution. *Optica* **3**, 1274 (2016).

[50] Kong, L. *et al.* Photonic integrated quantum key distribution receiver for multiple users. *Optics Express* **28**, 18449 (2020).

[51] Geng, W. *et al.* Stable quantum key distribution using a silicon photonic transceiver. *Optics Express* **27**, 29045 (2019).

[52] Sibson, P. *et al.* Chip-based quantum key distribution. *Nature Communications* **8** (2017).

[53] Paraïso, T. K. *et al.* A photonic integrated quantum secure communication system. *Nature Photonics* **15**, 850–856 (2021).

[54] Wei, K. *et al.* High-speed measurement-device-independent quantum key distribution with integrated silicon photonics. *Physical Review X* **10**, 031030 (2020).

[55] Yin, H.-L. *et al.* Measurement-device-independent quantum key distribution over a 404 km optical fiber. *Physical Review Letters* **117**, 190501 (2016).

[56] Zhou, X.-Y. *et al.* Reference-frame-independent measurement-device-independent quantum key distribution over 200 km of optical fiber. *Physical Review Applied* **15**, 064016 (2021).

[57] Zhou, L. *et al.* Experimental quantum communication overcomes the rate-loss limit without global phase tracking. *Physical Review Letters* **130**, 250801 (2023).

[58] Semenenko, H. *et al.* Chip-based measurement-device-independent quantum key distribution. *Optica* **7**, 238 (2020).

[59] Zhang, W. *et al.* A device-independent quantum key distribution system for distant users. *Nature* **607**, 687–691 (2022).

[60] Chen, J.-P. *et al.* Quantum key distribution over 658 km fiber with distributed vibration sensing. *Physical Review Letters* **128**, 180502 (2022).

[61] Zhou, L., Lin, J., Jing, Y. & Yuan, Z. Twin-field quantum key distribution without optical frequency dissemination. *Nature Communications* **14** (2023).
